# Association between relative fat mass and increased prevalence of gallstone disease in American adults: A cross-sectional study

**DOI:** 10.1097/MD.0000000000047790

**Published:** 2026-02-20

**Authors:** Yuwei Wang, Guanqun Hu, Shuyi Zhang

**Affiliations:** a Department of Gastroenterology II, Tianjin Union Medical Center, The First Affiliated Hospital of Nankai University, Tianjin, China; b Department of Neurology, Tianjin Union Medical Center, The First Affiliated Hospital of Nankai University, Tianjin, China.

**Keywords:** cross-section study, gallstone disease, NHANES, obesity, relative fat mass

## Abstract

The aim of this study was to investigate the relationship between relative fat mass (RFM) and the occurrence of gallstone disease (GSD) among American adults. A total of 15,560 adults were included. RFM, body mass index (BMI) and WC were taken into account. The receiver operating characteristic curve was utilized. Logistic regression and restricted cubic spline were employed to examine the potential correlation between RFM and the incidence of GSD. A cohort of 7515 individuals with GSD was studied. RFM, BMI, and WC were significantly associated with an elevated incidence of GSD in women in the unadjusted and adjusted models. Specifically, for every 1 standard deviation (SD) increase in BMI, WC, and RFM, the odds of GSD increased by 69%, 71%, and 77% in women. Notably, RFM had a higher area under the curve value compared to BMI in women. Among women, the prevalence of GSD was associated with a higher prevalence among individuals in the highest RFM tertile than in the lowest tertile (OR = 3.08, 95% confidence interval: 2.13–4.47, *P* < .001). The application of restricted cubic spline regression demonstrated a linear relationship between RFM and the likelihood of GSD. Higher RFM is positively associated with a higher prevalence of GSD in American women. Compared to BMI, RFM demonstrates a stronger association with GSD and may serve as a more useful anthropometric indicator in this population.

## 1. Introduction

Gallstones, crystalline deposits that frequently form in the gallbladder, are a prevalent condition among certain individuals. Notably, patients who are overweight and exhibit hypertriglyceridemia, a condition characterized by elevated blood triglyceride levels, are at a heightened risk for developing gallstone disease (GSD).^[1,2]^ Prior studies have delineated a comprehensive list of risk factors for GSD, which encompass female gender, metabolic syndrome, physical inactivity, obesity or overweight status, nonalcoholic fatty liver disease, and dietary factors such as excessive caloric intake, high glycemic load, inadequate fiber consumption, and high heme iron intake. Additionally, rapid weight loss stemming from bariatric surgery, prolonged fasting, and long-term reliance on total parenteral nutrition have also been identified as risk factors for GSD.^[3]^

Obesity is typically defined by the accumulation of excessive adipose tissue, having profound implications for an individual’s physical well-being.^[4]^ According to projections from the World Obesity Federation, it is anticipated that by 2035, over 4 billion people worldwide will be affected by obesity or being overweight, constituting a substantial portion exceeding half of the global population.^[5]^ Among the myriad of risk factors for gallstone disease (GSD), obesity has garnered significant attention. Prior studies have lent credence to the association between obesity and GSD.^[6,7]^ While body mass index (BMI) is commonly utilized as a metric for evaluating obesity, it is acknowledged that BMI alone is insufficient for accurately assessing obesity, as individuals with identical BMI values may exhibit varying body shapes and fat distribution patterns.^[8]^ In 2018, scientists introduced a novel obesity measurement, relative fat mass (RFM), which offers a more precise estimation of an individual’s total body fat proportion compared to BMI.^[9]^ RFM incorporates waist circumference (WC) and height to provide a more comprehensive assessment of fat mass. In this study, we aim to delve into the correlation between RFM and the prevalence of GSD. Furthermore, we also seek to evaluate and compare the performance of RFM, BMI, and waist circumference in assessing the prevalence of GSD.

## 2. Materials and methods

### 2.1. Study population

This cross-sectional study relied on data derived from the National Health and Nutrition Examination Survey (NHANES) from 2017 to 2020. The National Center for Health Statistics (NCHS), under the oversight of an ethics review board, gathered a diverse array of health-related data, encompassing demographic profiles, physical examination outcomes, and medical condition questionnaires. Prior to their participation, all participants provided written informed consent for their inclusion in the NHANES. The comprehensive dataset is publicly accessible on the NHANES website (http://www.cdc.gov/nchs/nhanes.htm). Prior to commencing data collection and NHANES health screenings, explicit informed consent was obtained from all eligible participants. Tianjin Union Medical Center review board has ascertained that the study in question is exempt from certain requirements, owing to its utilization of publicly accessible and deidentified data, thus negating the necessity for obtaining informed consent from participants. This study was deemed exempt by Tianjin Union Medical Center as it involved the analysis of publicly available data. Ethical content is available on the website (NHANES – NCHS Research Ethics Review Board Approval (cdc.gov)). The study adhered strictly to the Strengthening the Reporting of Observational Studies in Epidemiology (STROBE) guidelines to ensure rigor and transparency in its reporting. The study adheres to the Declaration of Helsinki.

To account for the complex, multistage probability sampling design of NHANES and to generate estimates that are representative of the noninstitutionalized US population, all analyses incorporated the appropriate survey examination weights, as recommended by the National Center for Health Statistics (NCHS).

**Figure 1.**
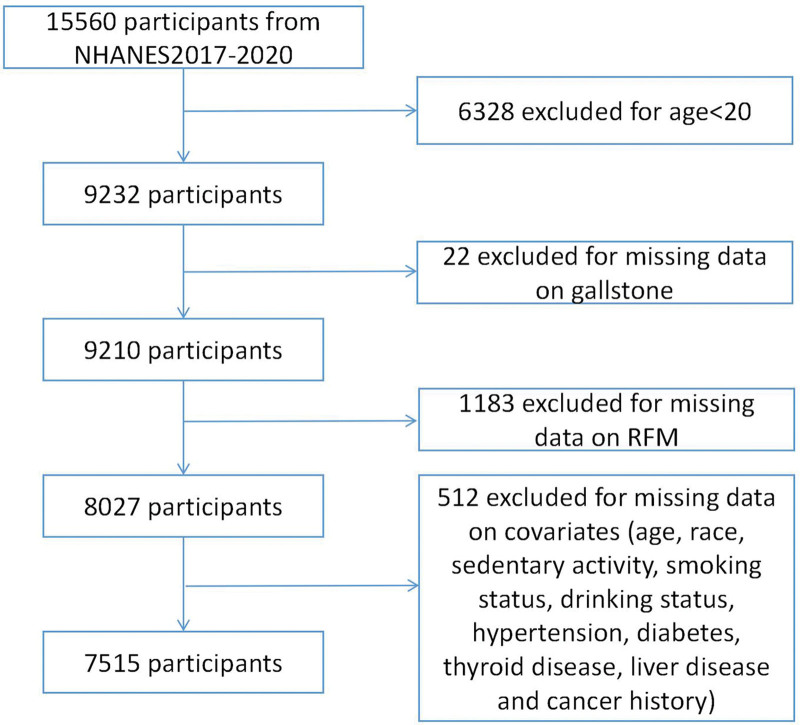
Flowchart of the sample selection process of the study population. RFM = relative fat mass.

Out of the initial 15,560 participants recruited during this period, we excluded individuals below 20 years of age (n = 6328), those lacking gallstone-related data (n = 22), participants with invalid RFM data (n = 1183), and those with incomplete covariate information (n = 512). Consequently, a total of 7515 subjects were included in the final analysis, as depicted in Figure 1.

### 2.2. RFM, BMI, and WC

The RFM is calculated by WC, height and sex. RFM = 64 − (20 × height/WC) + (12 × sex), where sex is a sex-specific constant set to 0 for men and 1 for women. Measurements of height and WC were obtained by a qualified health professional in the Mobile Examination Center (MEC). The MEC was equipped with a dedicated height-measuring apparatus, ensuring precise results. Participants were instructed to stand barefoot, with their backs firmly pressed against the measuring board and their heads positioned at a horizontal level, to guarantee the utmost accuracy. For the calculation of WC, the measurement was taken at the point just above the iliac crest, along the mid-axillary line. Special care was taken to ensure the readings were accurate to 0.1 cm, taken at the end of a normal respiratory cycle.

Furthermore, BMI was also calculated, utilizing the participant’s weight and height. Given the inherent differences in RFM, BMI, and WC among various gender groups, distinct calculations were carried out separately for men and women.

### 2.3. GSD diagnosis

GSD is dependent on a patients’ self-report response to the question: “Has a doctor or other health professional ever told you that you had gallstones?.” The simple and easy method has been employed in prior research studies.^[10]^

### 2.4. Covariates

A range of potential covariates were evaluated based on prior research,^[11,12]^ encompassing demographic factors such as age and race/ethnicity, lifestyle habits like sedentary behavior and smoking/drinking status, as well as preexisting medical conditions including hypertension, diabetes, thyroid disease, liver diseases, and cancer history. The racial/ethnic categories encompassed non-Hispanic whites, non-Hispanic blacks, Mexican–Americans, and other Hispanics, with all remaining categories classified as “other.”

According to the literature definitions,^[13]^ respondents were classified as nonsmokers if they had smoked fewer than 100 cigarettes in their lifetime, and smokers if they had smoked 100 or more cigarettes. Sedentary status was determined if participants reported engaging in 600 or more minutes of sedentary activity daily, whereas non-sedentary status was ascribed to those engaging in <600 minutes of sedentary activity daily. Alcohol drinking status was stratified into 3 categories: nondrinkers (never consumed alcohol), light drinkers (consumed no alcohol or 1 drink per day in the past year), and heavy drinkers (consumed 2 or more drinks per day in the past year). Preexisting medical conditions were evaluated through a questionnaire that inquired about blood pressure, diabetes, liver disease, thyroid disease, and cancer history. A diagnosis of hypertension was made if participants reported being told they had high blood pressure on 2 or more occasions. Self-reported responses were utilized to define diabetes, thyroid disease, liver diseases, and cancer history.

### 2.5. Statistical analysis

The participants’ characteristics were represented using percentages for categorical variables, and either means accompanied by standard deviations (SD) or medians with interquartile ranges for continuous variables, depending on the specific context. To evaluate differences in continuous data, the *t*-test was employed, while categorical data were analyzed using the chi-square (χ^2^) test. To further examine disparities among groups, we conducted a 1-way analysis of variance (ANOVA) for normally distributed variables, the Kruskal–Wallis test for variables exhibiting a skewed distribution, and the chi-square (χ^2^) test for categorical variables. Additionally, a receiver operating characteristic (ROC) curve, inclusive of the area under the curve (AUC) and its 95% confidence interval (CI), was utilized to assess the capacity of the RFM, BMI, and WC in identifying GSD. To statistically compare the discriminatory performance among these anthropometric indices, the differences between the AUC values were assessed using DeLong’s test for 2 correlated ROC curves.

To investigate the association between RFM, BMI, WC, and the prevalence of GSD, we employed logistic regression models to estimate the odds ratios (ORs) and corresponding 95% CIs. These ORs quantify the change in the odds of GSD for every 1-SD increment in the adiposity indicators. Our analyses encompassed 3 progressively adjusted models: model 1 incorporated sociodemographic factors such as age and race; model 2 further accounted for sedentary activity, smoking status, and drinking status in addition to model 1; and model 3 extended model 2 by including hypertension, diabetes, liver diseases, thyroid disease, and cancer history. To explore the dose-response relationship between GSD and RFM, we utilized restricted cubic splines. Finally, we performed a multivariable logistic regression analysis, examining RFM as both a continuous variable and a categorical variable, stratified into tertiles, to comprehensively assess its influence on GSD prevalence.

The assessment of the association between RFM and GSD incorporated various variables, encompassing race, age categories (20–60 years, ≥60 years), sedentary activity, smoking and drinking habits, as well as medical histories of hypertension, diabetes, thyroid disease, liver diseases, and cancer. To quantify subgroup heterogeneity and investigate potential interactions between subgroups and RFM, we utilized multivariate logistic regression models, coupled with likelihood ratio testing.

For the statistical analyses, we employed the Free Statistics software (version 1.9.1) developed by Beijing Free Clinical Medical Technology Co., Ltd. in Beijing, China. A 2-sided *P*-value of <.05 was deemed indicative of statistical significance. The data analysis was conducted in May 2024.

## 3. Results

### 3.1. Baseline characteristics

The baseline characteristics of the participants with and without GSD are shown in Table 1. A total of 7515 (10.5%) subjects with GSD were enrolled. The average age of the participants was 50.5 years, and 3805 (50.6%) individuals were women. RFM in participants with GSD was 33.5 ± 8.7 and 29.6 ± 7.1 in participants without GSD.

**Table 1:** Baseline characteristics of the participants with and without GSD.

Variables	Total (n = 7515)	Without GSD n = 6737(89.6%)	With GSD n = 778(10.4%)	*P*-value
Age (yr)	50.5 ± 17.3	49.7 ± 17.3	57.8 ± 15.6	<.001
Gender, n (%)				<.001
Men	3710 (49.4)	3486 (51.7)	224 (28.8)	
Women	3805 (50.6)	3251 (48.3)	554 (71.2)	
Race, n (%)				<.001
Non-Hispanic White	2672 (35.6)	2332 (34.6)	340 (43.7)	
Non-Hispanic Black	1988 (26.5)	1831 (27.2)	157 (20.2)	
Mexican American	878 (11.7)	776 (11.5)	102 (13.1)	
Other	1977 (26.3)	1798 (26.7)	179 (23)	
BMI, kg/m^2^	30.0 ± 7.3	29.6 ± 7.1	33.5 ± 8.6	<.001
WC, cm	101.1 ± 17.2	100.2 ± 16.9	108.8 ± 17.5	<.001
RFM	36.2 ± 8.8	35.5 ± 8.7	41.7 ± 8.0	<.001
Smoking status, n (%)				.006
Nonsmoker	3160 (42.0)	2797 (41.5)	363 (46.7)	
Smoker	4355 (58.0)	3940 (58.5)	415 (53.3)	
Drinking status, n (%)				<.001
Nondrinker	678 (9.0)	609 (9)	69 (8.9)	
Light drinker	3433 (45.7)	2999 (44.5)	434 (55.8)	
Heavy drinker	3404 (45.3)	3129 (46.4)	275 (35.3)	
Sedentary activity, n (%)				.063
Sedentary	1134 (15.1)	999 (14.8)	135 (17.4)	
Non-sedentary	6381 (84.9)	5738 (85.2)	643 (82.6)	
Diabetes, n (%)				<.001
Yes	1111 (14.8)	916 (13.6)	195 (25.1)	
No	6404 (85.2)	5821 (86.4)	583 (74.9)	
Hypertension, n (%)				<.001
Yes	2309 (30.7)	1944 (28.9)	365 (46.9)	
No	5206 (69.3)	4793 (71.1)	413 (53.1)	
Thyroid disease, n (%)				<.001
Yes	865 (11.5)	701 (10.4)	164 (21.1)	
No	6650 (88.5)	6036 (89.6)	614 (78.9)	
Cancer history, n (%)				<.001
Yes	787 (10.5)	646 (9.6)	141 (18.1)	
No	6728 (89.5)	6091 (90.4)	637 (81.9)	
Liver disease, n (%)				<.001
Yes	379 (5.0)	304 (4.5)	75 (9.6)	
No	7136 (95.0)	6433 (95.5)	703 (90.4)	

BMI = body mass index, GSD = gallstone disease, RFM = relative fat mass, WC = waist circumference.

### 3.2. Relationship between standardized adiposity indicators and GSD

A positive association was detected between RFM, BMI, WC and the prevalence of GSD in the non-adjusted crude model in women group and remain stable in each adjusted model. Further adjustment did not significantly affect the results, after adjusting for age, race, sedentary activity, smoking status, drinking status, hypertension, diabetes, liver diseases, thyroid disease, and cancer history in model 3. The odds of GSD in women increased by 69 % (OR = 1.69, 95% CI: 1.56–1.82, *P* < .001), 71 % (OR = 1.71, 95% CI: 1.51–1.94, *P* < .001) and 77% (OR = 1.77, 95% CI: 1.53–2.04, *P* < .001) for each 1-SD increase in BMI, WC, and RFM respectively (Table 2).

**Table 2:** Association of standardized adiposity indicators and GSD among men and women.

Exposure	N	Non-adjusted model		Model1		Model2		Model 3	
		OR (95% CI)	*P*-value	OR (95% CI)	*P*-value	OR (95% CI)	*P*-value	OR (95% CI)	*P*-value
Men	3710								
WC (1-SD)		1.45 (1.19–1.77)	<.001	1.37 (1.07–1.76)	0.015	1.36 (1.05–1.77)	.024	1.22 (0.91–1.65)	.16
BMI (1-SD)		1.26 (1.04–1.52)	.02	1.36 (1.08–1.70)	0.01	1.35 (1.06–1.71)	.017	1.22 (0.94–1.59)	.121
RFM (1-SD)		1.63 (1.31–2.03)	<.001	1.43 (1.08–1.88)	0.014	1.41 (1.06–1.89)	.022	1.25 (0.92–1.71)	.143
Women	3805								
WC (1-SD)		1.78 (1.58–2.00)	<.001	1.8 (1.58–2.04)	<0.001	1.76 (1.55–2.00)	<.001	1.71 (1.51–1.94)	<.001
BMI (1-SD)		1.66 (1.51–1.82)	<.001	1.76 (1.61–1.93)	<0.001	1.74 (1.60–1.89)	<.001	1.69 (1.56–1.82)	<.001
RFM (1-SD)		1.94 (1.69–2.22)	<.001	1.87 (1.61–2.16)	<0.001	1.83 (1.58–2.12)	<.001	1.77 (1.53–2.04)	<.001

Model 1 adjusted for age, race.

Model 2 further adjusted for sedentary activity, smoking status, and drinking status plus model 1.

Model 3 further adjusted for hypertension, diabetes, thyroid disease, liver disease and cancer history plus model 2.

CI = confidence interval, BMI = body mass index, GSD = gallstone disease, OR = odds ratio, RFM = relative fat mass, WC = waist circumference.

### 3.3. ROC analysis

The logistic regression analysis yielded results indicating that RFM possesses independent predictive capacity for the onset of GSD. To compare its predictive prowess with traditional anthropometric indicators, namely BMI and WC, an ROC analysis was conducted.

**Figure 2.**
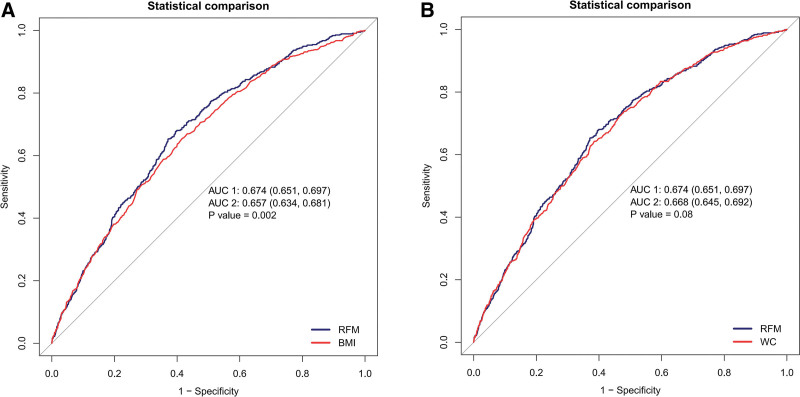
Receiver operating characteristic curves of RFM, BMI, WC for incident GSD. (A): RFM and BMI, (B) RFM and WC. BMI = body mass index, GSD = gallstone disease, RFM = relative fat mass, WC = waist circumference.

In women, RFM demonstrated a significantly superior discriminatory ability compared to BMI, as evidenced by a higher AUC value in both DeLong’s test and Bonferroni-adjusted comparisons (*P* = .002). However, no significant difference in AUC was observed between RFM and WC (DeLong’s test and Bonferroni-adjusted, *P* = .08), as depicted in Figure 2.

### 3.4. Relationship between RFM and GSD

The prevalence of GSD increased as the RFM increased, and the OR of tertile 3 was significantly higher than tertile 1 in women in the non-adjusted crude model (OR = 4.10, 95% CI: 2.85–5.88, *P* < .001). Further adjustment did not significantly affect the results, after adjusting for age, race, smoking status, drinking status, hypertension, diabetes, liver diseases, thyroid disease, and cancer history in model 3 (OR = 3.08, 95% CI: 2.13–4.47, *P* < .001); Table 3).

**Table 3:** Association between RFM and GSD in women.

Exposure	N	Non-adjusted model		Model1		Model2		Model 3	
		OR (95% CI)	*P*-value	OR (95% CI)	*P*-value	OR (95% CI)	*P*-value	OR (95% CI)	*P*-value
RFM (Continuous)	3805	1.12 (1.09–1.15)	<.001	1.11 (1.08–1.14)	<.001	1.11 (1.08–1.14)	<0.001	1.10 (1.08–1.13)	<.001
Categorical of RFM									
Tertile 1 (22.7–40.8)	1268	1 (Reference)		1 (Reference)		1 (Reference)		1 (Reference)	
Tertile 2 (40.8–45.9)	1268	2.23 (1.52–3.26)	<.001	1.89 (1.27–2.83)	.004	1.86 (1.23–2.80)	0.006	1.76 (1.13–2.75)	.018
Tertile 3 (45.9–58.2)	1269	4.10 (2.85–5.88)	<.001	3.51 (2.43–5.08)	<.001	3.37 (2.34–4.86)	<0.001	3.08 (2.13–4.47)	<.001
P for trend			<.001		<.001		<0.001		<.001

Model 1 adjusted for age, race.

Model 2 further adjusted for sedentary activity, smoking status, and drinking status plus model 1.

Model 3 further adjusted for hypertension, diabetes, thyroid disease, liver disease and cancer history plus model 2.

CI = confidence interval, GSD = gallstone disease, OR = odds ratio, RFM = relative fat mass.

**Figure 3.**
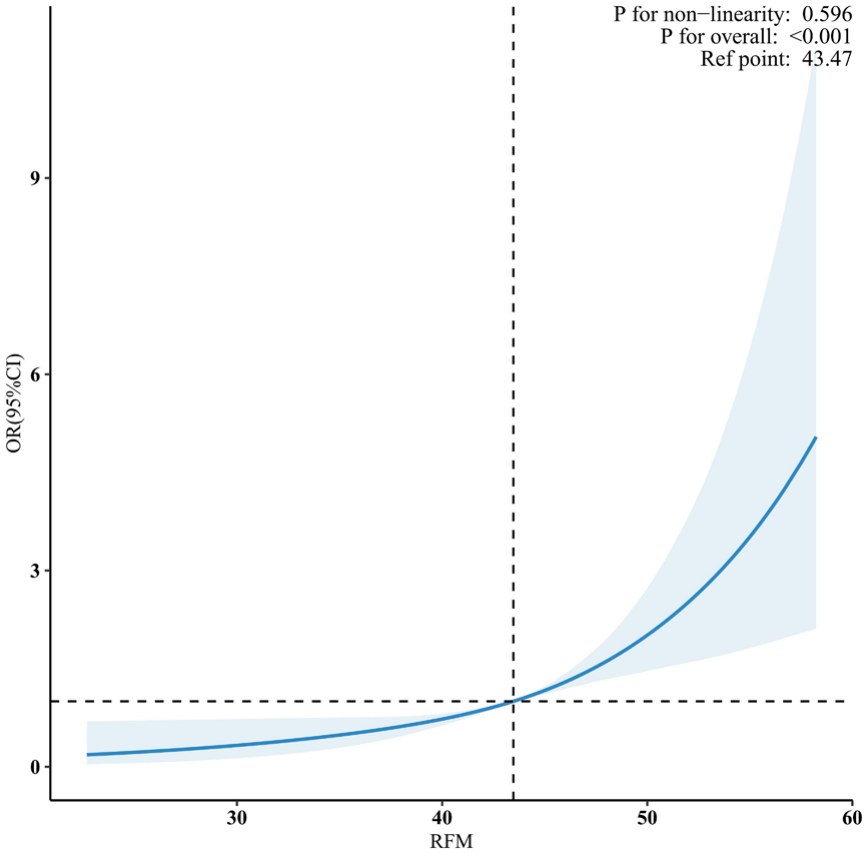
The relationship between RFM and GSD among women. CI = confidence interval, GSD = gallstone disease, OR = odds ratio, RFM = relative fat mass.

Figure 3 illustrates the findings of the restricted cubic spline analysis, which was performed to assess the association between RFM and the prevalence of GSD in women. This analysis revealed a linear relationship between RFM and the probability of GSD, which remained significant even after accounting for potential confounding factors (nonlinearity: *P* = .596; Fig. 3).

### 3.5. Subgroup analysis between GSD and the relative fat mass among women

A comprehensive and rigorous evaluation was conducted to determine potential variations in the association between RFM and the prevalence of GSD. This analysis encompassed a range of variables, such as age categories (20–60 years, ≥60 years), ethnicity, smoking habits, alcohol consumption, sedentary lifestyle, hypertension, diabetes, liver conditions, thyroid disorders, and a prior cancer history.

**Figure 4.**
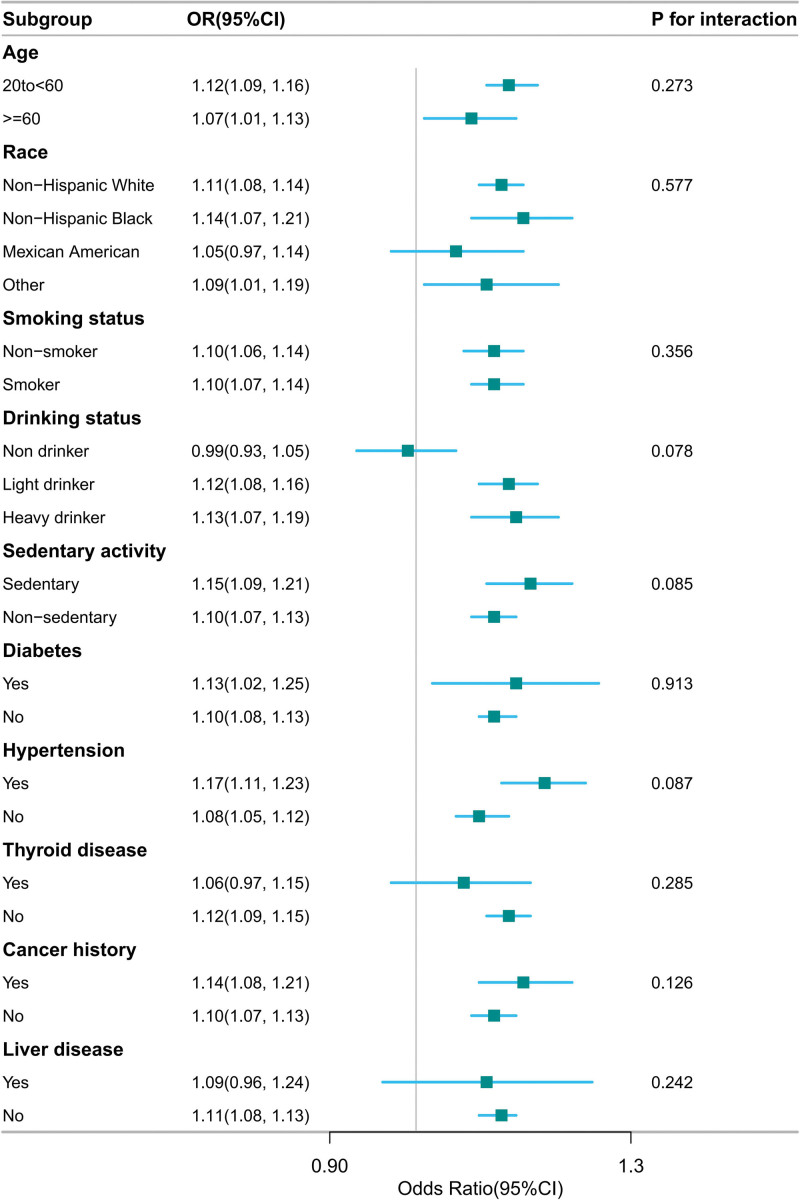
Subgroup analysis between GSD and the relative fat mass among women. Adjusted for age, race, smoking status, drinking status, sedentary activity, diabetes, hypertension, thyroid disease, liver diseases and cancer history. CI = confidence interval, GSD = gallstone disease, OR = odds ratio.

To assess the heterogeneity across various subgroups, a multivariate logistic regression analysis was utilized, while the likelihood ratio test was employed to scrutinize the interactions between subgroups and RFM. Notably, no significant interaction effects were observed in women (Fig. 4).

All sensitivity analyses confirmed that the positive association between RFM and GSD in women remained statistically significant. This includes, but is not limited to, models with further adjustment for potential confounders, alternative modeling of RFM (as continuous or tertile variable), and and subgroup evaluations across different populations.

## 4. Discussion

The present study found that the significant positive associations of RFM and BMI with GSD in a nationally representative sample of US adults were exclusive to women. Across both non-adjusted and adjusted models, the OR for RFM in the third tertile was substantially higher than that in the first tertile for women. To our knowledge, our study pioneers in examining the relationship between RFM and GSD. Notably, RFM demonstrated superior discriminatory ability for GSD compared to BMI among women. In ROC analysis, RFM outperformed BMI in identifying GSD. Our findings reveal a positive linear association between RFM and the probability of GSD for women, which persisted after adjustment for potential confounding factors. This association was consistent across all examined subgroups, with no significant interactions observed.

Obesity has been consistently established as the main risk factor for the development of GSD,^[14]^ The established pathophysiological link between obesity and gallstone formation lies in the phenomenon of biliary cholesterol supersaturation.^[15]^ This condition is mediated by enhanced activity of HMG-CoA reductase, leading to increased hepatic cholesterol secretion.^[16]^ In individuals with obesity, there is oversecretion of biliary cholesterol, bile salts, and phospholipids. However, the secretion rate of cholesterol exceeds that of the other biliary lipids, thereby resulting in cholesterol-supersaturated bile.^[16]^ BMI was thought as an estimate of the general obesity.^[17]^ Previous studies showed BMI as an independent risk factor of GSD.^[7,18]^ However, the usefulness of this analysis is controversial because the regional distribution of body fat, rather than the total quantity, is more crucial for the formation of gallstones.^[15]^ Research indicates that visceral adipose tissue has a stronger correlation with metabolic disorders than subcutaneous fat.^[19,20]^ The possible pathophysiological mechanism of GSD is associated with the increase in plasma insulin levels. Similarly, elevated levels of leptin also have been associated with a higher diagnosis of GSD.^[14]^ Central abdominal fat has been mostly associated with insulin resistance with the consequent increase in the hepatic cholesterol secretion; contributing as one of the multiple mechanisms associated with the development of gallstones.^[14]^ The fact that RFM’s association with GSD remained significant after adjusting for diabetes status in our analysis suggests that RFM may capture elements of obesity-related metabolic dysfunction, such as subclinical insulin resistance, that are not fully accounted for by clinical diagnoses and are not adequately reflected by BMI.

BMI was initially formulated by a Belgian mathematician approximately 200 years ago and it currently serves as the most commonly utilized diagnostic instrument for obesity. However, it is insufficient in distinguishing between lean and fat mass, as well as visceral and subcutaneous fat.^[21]^ Previous study showed that elevated BMI, WC and waist-to-hip ratio (WHR) were independent risk factors for new-onset GSD.^[7,22]^ Moreover, higher weight-adjusted-waist index (WWI) levels also connected with increased prevalence of gallstones.^[23]^ In present study, we chose RFM as our key indicators which included BMI as well as WC. RFM showed superiority over BMI. RFM is an innovative obesity index that was introduced by researchers in the United States in 2018, offering a more precise estimation of total body fat percentage in contrast to BMI.^[9]^ Therefore, RFM was regarded as an abdominal obesity criterion for metabolic syndrome.^[24]^ According to a survey conducted in South Korea, the RFM misclassification rate was found to be lower than that of BMI in estimating body adiposity in women participants.^[25]^ RFM is now widely used in different fields, which has been associated with a number of diseases including venous thromboembolism,^[26]^ coronary heart disease,^[27]^ hypertension^[28]^ and type 2 diabetes.^[29]^ In the present study, RFM serves as a superior surrogate marker for visceral adiposity compared to the traditional indicator BMI, making it a better predictor of GSD prevalence.

Consistent with previous report,^[30]^ we observed a marked gender disparity, with a stronger obesity-gallstone association in women. Beyond the established role of sex hormones, the biological relevance of RFM itself may differ by sex-specific constant. RFM, calculated as 64 − (20 × height/WC) + (12 × sex), inherently incorporates a sex-specific constant, acknowledging fundamental physiological differences in body composition. Women typically have a higher percentage of body fat and a different fat distribution pattern (more subcutaneous and gluteofemoral fat) compared to men for the same BMI. The RFM formula, heavily reliant on waist circumference adjusted for height, might thus be capturing a particularly pathogenic fat distribution phenotype in women – one that closely aligns with visceral adiposity even within lower BMI ranges.^[31]^ This visceral fat is metabolically active, promoting insulin resistance and dyslipidemia, key drivers of cholesterol-supersaturated bile. In men, fat distribution patterns and their metabolic consequences may be less specifically captured by the WC/height ratio alone, or other unmeasured factors (e.g., dietary patterns, alcohol consumption) might play a more dominant role in GSD pathogenesis, potentially diluting the specific signal from RFM.^[32]^ Therefore, RFM may not only be a better indicator of fat mass but also a more sensitive proxy for the metabolically adverse fat distribution that is critically involved in gallstone formation in women. This pattern strongly implicates the role of sex-specific hormonal factors. The underlying mechanism is likely closely related to the mediating role of sex hormones. Estrogen, via its membrane receptor GPR30, promotes cholelithogenesis through a dual hit mechanism: it induces cholesterol-supersaturated bile by disrupting the bile acid synthesis pathway, and simultaneously creates a pro-lithogenic environment by inhibiting gallbladder emptying.^[33]^Moreover, symptomatic gallstones are more prevalent during pregnancy. Their formation is likely attributed to hormone-induced alterations in bile composition conducive to stone formation, coupled with reduced gallbladder motility.^[34]^

Our limitations necessitate additional exploration. The cross-sectional nature of this study precludes the establishment of a causal relationship between RFM and GSD. The diagnosis of GSD relied solely on self-reported physician diagnosis, which is susceptible to recall bias and likely underestimates the prevalence of asymptomatic gallstones. The study did not account for certain important risk factors for GSD, notably a history of rapid weight loss (through diet, medication, or bariatric surgery). By relying on clinical diagnosis, this study could not identify or include individuals with asymptomatic (silent) gallstones. What is more, for certain subgroup analyses (e.g., by race), the sample sizes were relatively small, which may limit statistical power and result in less stable estimates of the odds ratios (ORs). Therefore, the interpretation of findings for these specific subgroups should be made with caution. Despite these limitations, our study benefits from a large, nationally representative sample, rigorous weighting methods, and consistent findings across multiple sensitivity analyses, which strengthen the validity of the observed associations.

## 5. Conclusions

In summary, our study indicates that RFM, an easily calculable index incorporating waist circumference, shows a stronger association with GSD among women individuals compared to BMI. The pronounced association in women further highlights the important interplay between body fat distribution and sex-specific hormonal physiology in GSD pathogenesis. These findings suggest that RFM may be a more relevant anthropometric indicator for GSD than BMI. If confirmed by future longitudinal or interventional studies, RFM assessment could guide more personalized risk stratification and inform the comprehensive management of obesity in women with higher prevalence of GSD. Furthermore, elucidating the precise mediating roles of metabolic and hormonal factors remains a crucial direction for future research.

## Acknowledgments

We acknowledge all of the participants and staff involved in NHANES for their valuable contributions.

## Author contributions

**Data curation:** Yuwei Wang.

**Funding acquisition:** Guanqun Hu.

**Methodology:** Yuwei Wang.

**Supervision:** Guanqun Hu, Shuyi Zhang.

**Validation:** Guanqun Hu.

**Writing – original draft:** Yuwei Wang.

**Writing – review & editing:** Guanqun Hu, Shuyi Zhang.
